# Aggressive behaviours, food deprivation and the *foraging* gene

**DOI:** 10.1098/rsos.170042

**Published:** 2017-04-26

**Authors:** Silu Wang, Marla B. Sokolowski

**Affiliations:** 1 Department of Zoology, University of British Columbia, 6270 University Boulevard, Vancouver, British Columbia, CanadaV6T 1Z4; 2 Department of Ecology and Evolutionary Biology, University of Toronto, 25 Willcocks Street, Toronto, Ontario, CanadaM5S 3B2; 3 Child and Brain Development Program, Canadian Institute for Advanced Research (CIFAR), 180 Dundas Street West, Suite 1400, Toronto, Ontario, CanadaM5G 1Z8

**Keywords:** pleiotropy, foraging, aggression, food deprivation, escalation, modular

## Abstract

A pleiotropic gene governs multiple traits, which might constrain the evolution of complexity due to conflicting selection on these traits. However, if the pleiotropic effect is modular, then this can facilitate synergistic responses to selection on functionally related traits, thereby leveraging the evolution of complexity. To understand the evolutionary consequence of pleiotropy, the relation among functionally different traits governed by the same gene is key. We examined a pleiotropic function of the *foraging* (*for*) gene with its rover and sitter allelic variants in fruit fly, *Drosophila melanogaster*. We measured *for*'s effect on adult male aggressive behaviours and whether this effect was shaped by *for*'s known role in food-related traits. Rover exhibited higher levels of offensive behaviour than sitters and s2, a sitter-like mutant on rover genetic background. With a Markov chain model, we estimated the rate of aggression escalation, and found that the rover pattern of aggressive escalation more rapidly intensified fights. Subsequent analysis revealed that this was not caused by *for*'s effect on food-related traits, suggesting that *for* might directly regulate aggressive behaviours. Food deprivation did not elevate aggression, but reduced intermediate-level aggressive behaviours. Aggression and other foraging-related behaviour might comprise a synergistic trait module underlaid by this pleiotropic gene.

## 1. Introduction

Pleiotropic genetic effects were proposed to modulate multiple traits due to a mutation at a single locus [1]. The effect was thought to be ‘universal’, such that each mutation had the potential to influence all the traits [2]. Universal pleiotropy constrains adaptation and evolution of complexity, especially if the beneficial change in one trait coincides with the deleterious change of another trait (cost of complexity) [2,3]. However, if a pleiotropic effect is ‘modular’, it is confined to a trait module (consists of functionally/developmentally similar traits) and facilitates the synergistic response to selection among traits, allowing the evolution of complexity [4]. There have been many modelling and genomic studies investigating the distribution of pleiotropic gene effects and the number of traits under control, which have not yet reached an agreement on which model is more biologically applicable [5–8].

Further empirical examination of the linkage between genotype and concrete phenotypes, and the functional association among phenotypes is necessary. Evaluating functional relations among phenotypes allows us to understand the functional assembly of phenotypes, providing a blueprint for molecular-level investigation of pleiotropy. For the phenotypes that confer drastically different functions or are under distinct selections (e.g. natural versus sexual selection), investigating trait–trait association reveals pleiotropic architecture underlying coevolution of these traits. Compiling trait–gene and trait–trait functional relations is essential to understand the proximate functions and ultimate fitness consequences of pleiotropy. However, concurrent examinations of gene–trait and trait–trait associations are still insufficient.

A pleiotropic gene, *for*, provides a great opportunity to concurrently investigate trait–trait functional relations in addition to gene–trait causal effects. A single-locus genetic variation in this cGMP-dependent protein kinase (PKG) gives rise to rover and sitter allelic polymorphism in natural fruit fly populations [9–13]. This polymorphism is characterized by diagnostic differences in different metabolic and behavioural traits (reviewed in [14]), such as larval foraging path length, adult behavioural and physiological responses to food deprivation [15], learning and memory, sleeping patterns and stress responses [16]. The polymorphism has been maintained by negative frequency-dependent selection in the laboratory [17]. However, the functional architecture among these phenotypes remains to be investigated.

One potential interaction among *for*-regulated traits lies in the motivation state, the condition that causes an animal to pursue a certain goal [18]. The *for* gene modulates the motivation state through energy homeostasis [15,19]. Specifically, after acute food deprivation, haemolymph sugar levels decreased further, while food intake increased less in rover larvae than in sitter larvae. In addition, after 24 h of food deprivation, adult rovers show greater sucrose responsiveness, indicating greater levels of hunger in rovers than in sitters [20]. The variation in hunger state between rover and sitter may predispose them to different motivational states of aggression. Motivational states, such as hunger, have been shown to be important estimators of aggressive behaviour in many species [21–23] and can thus be the central mediator through which *for* affects aggression.

Food deprivation can be a short-term trigger for aggressive behaviour. When food is scarce, aggression levels often increase to defend or acquire food resources [24–26]. Game theory predicts increased use of fighting when the value of food increases, due to increased resource quality and/or internal physiological conditions actuated by food deprivation [23]. Empirically, limited feeding opportunity or food deprivation increases the frequency of offensive behaviour [27–29], causes shifts in territorial behaviour [30] and enhances the chance of victory [31,32] in various invertebrates and vertebrates. Thus, standing difference in hunger state arising from the *for* polymorphism might cause aggression differences.

Here, we investigate the effect of *for* on aggression and its response to food deprivation. If the *for* polymorphism has an effect on aggressive behaviour, rovers and sitters should demonstrate aggression differences in the frequency of behaviour and/or the pattern of aggressive escalations (**H1**). If the relation between *foraging* and aggression is established through the differential food deprivation response in rovers and sitters, this relationship should be reshaped by food deprivation (**H2**). For instance, if the lower hunger state of sitters imposes less aggressive motivation than rovers, food deprivation should elevate the aggressive behaviour of sitters and minimize or erase the aggression difference between strains. Testing these hypotheses will facilitate the understanding of a pleiotropic gene and its functional architecture in targeting the trait complex.

## 2. Material and methods

### 2.1. Strains

The wild-type rover *for*^*R*^ and *for*^*s*^ strains differ in their second pair of chromosomes where the *foraging* gene resides but share their X and third pairs of chromosomes [10]. We also used the s2 strain, which is a sitter mutant generated on a rover genetic background to control for the backgrounds and detect aggression difference that are due to *for* [10,33]. Test flies were reared at 25 ± 1°C and 12 L : 12 D photo-cycle with lights on at 08.00 h. Newly hatched larvae of the same strain were collected and grown in groups of 20–25 larvae maintained in 50 ml plastic culture vials plugged with a sponge. Each vial contained 10 ml of standard culture medium described below. Before eclosion, male pupae were isolated in individual glass vials (10 × 75 mm, with 1 ml food in each vial) and plugged with a cotton ball. These vials were left undisturbed until the food deprivation treatment. The standard culture medium contained: 50 g Baker's yeast; 100 g sucrose; 16 g agar; 0.1 g KPO_4_; 8 g KNaC_4_H_4_O_6_·4H_2_O; 0.5 g NaCl; 0.5 g MgCl_2_ and 0.5 gFe_2_(SO_4_)_3_ per litre of tap water.

### 2.2. Aggression trials

Adult male flies were tested at 5 ± 1 days in age. Video recording took place from 9.30 and 14.00 on each day. The observation chamber was made of a circular hollow cap (inner diameter = 19 mm, inner height = 14 mm) covered by micro cover glass (22 × 22 mm). To optimize the level of encounters, a drop of yeast paste (approx. 2 µl) was placed on the centre of the food surface [34]. The inner food cup diameter was 10 mm and the fluon was applied on the chamber wall, so that subjects would remain either on the floor or on food [35].

Five minutes of acclimatization were allowed before the 10 min behavioural scoring period. Within an arena, the two subjects of the same strain and treatment were recorded as one unit in terms of the frequency of behaviours, thus the behavioural variables reflect the intensity of aggression of the pair. We identified behaviours involved in aggression according to the male fruit fly aggression ethogram [36]. The nine behaviours under study included both offensive behaviours: head-to-head interaction (boxing, tussling and holding), lunging, offensive wing threat, offensive fencing and chasing; and defensive behaviours: defensive wing threat, defensive fencing, approach and retreat. We recorded the total frequency of each behaviour displayed by the pair. The mean body weights of the subjects were measured for each strain and treatment after the behaviour trials.

### 2.3. Food deprivation treatment

One day before the aggression assay, half of the flies of each strain were randomly selected for the deprivation treatment. They were moved into an empty glass vial (same dimension) with 1 ml of agar for the food deprivation treatment. The agar in the food deprivation treatment provided hydration for the flies. The fed flies were moved into a new vial with regular food to control for handling effect. Fed or food-deprived intra-strain pairs of rover, sitter or s2 (six combinations: *n* = 11 for deprived rover–rover, *n* = 10 for fed rover–rover, *n* = 11 for deprived sitter–sitter, *n* = 11 for fed sitter–sitter, *n* = 11 for deprived s2–s2 and *n* = 12 fed s2–s2) were introduced simultaneously into each fighting chamber. The loading sequence and locations of the six arenas were randomized among strain–treatment combinations prior to filming the encounters. We also measured the total number of times that both flies moved onto the food cup as an activity score under each treatment.

### 2.4. Aggressive escalation sequence analysis

The pattern of escalation of aggression could reflect the aggressive motivation states of the subjects. Based on an aggression sensitization effect model, or a warm-up effect model, in which the intensity of attack increases as the fight progresses [37–39], an absorbing Markov chain modelling was applied for social behavioural sequence analysis [40]. Under the warm-up effect model, escalation intensity increases in aggressive interactions as the higher-level aggressive behaviours replace lower-level aggressive behaviours. We modelled the warm-up effect as an absorbing Markov chain [40], in which various sequential behavioural transitions occur between an initial approach to the highly escalated ‘aggressive absorbing state’: head-to-head interaction (figure 2*a–c*). We recorded the pairwise directional transitions among all of the behaviours so as to calculate the probability of each transition from the preceding to succeeding behaviour as the frequency of a directional transition divided by the total frequency of all the transitions after the preceding behaviour. With the directional transition probability matrix [40], we first calculated the mean number of behavioural transitions that occurred between an initial approach to a head-to-head interaction. Then, we estimated the aggressive *escalation rate* as the reciprocal of the mean number of transitions from an initial approach to head-to-head interactions (figure 2*a–c*). We did escalation rate estimation for each pair of male opponents under each treatment. Only a subset of the fighting pairs in each strain and treatment (deprived rover: *n* = 6; fed rover: *n* = 6; deprived sitter: *n* = 10; fed sitter: *n* = 7; deprived s2: *n* = 6; fed s2: *n* = 6) exhibited at least four out of the nine aggressive behaviours used to generate each transition probability matrix for escalation rate estimation. Figure 2*a–c* shows the average weighted network for each strain Custom R code [41] for absorbing Markov chain modelling can be found on github.

### 2.5. Statistical method

The frequency of each aggressive behaviour observed during the scoring period is Poisson-distributed. Generalized linear model (GLM) with the Poisson error distribution was used to test the effect of strain and food deprivation treatments on total activity score and frequency of aggressive behaviours. *χ*^2^ analysis of deviance test followed by the Bonferroni correction [42] was exploited to examine the variation of aggressive behaviours among food deprivation treatments and strains in the GLM models. Principle component analysis (PCA) of offensive versus defensive behaviours followed by two-way analysis of variance (ANOVA) was conducted to collectively examine variation of offensive or defensive behaviours (first and second principle components, PC1 and PC2) among treatments and strains. Two-way ANOVA was used to test body weight variation among strains and treatments. We used R [43] for all the analyses.

Because of the small and uneven sample size, the variation in escalation rates among strains and treatments was investigated by bootstrapping (*n* = 10 000) transition rate differences among rover, sitter and s2 as well as between fed and food-deprived flies, followed by 95% confidence interval evaluation of significance. The raw data [44] of all the analyses were deposited in Dryad.

## 3. Results

### 3.1. *Foraging* and aggression

**Figure 1.**
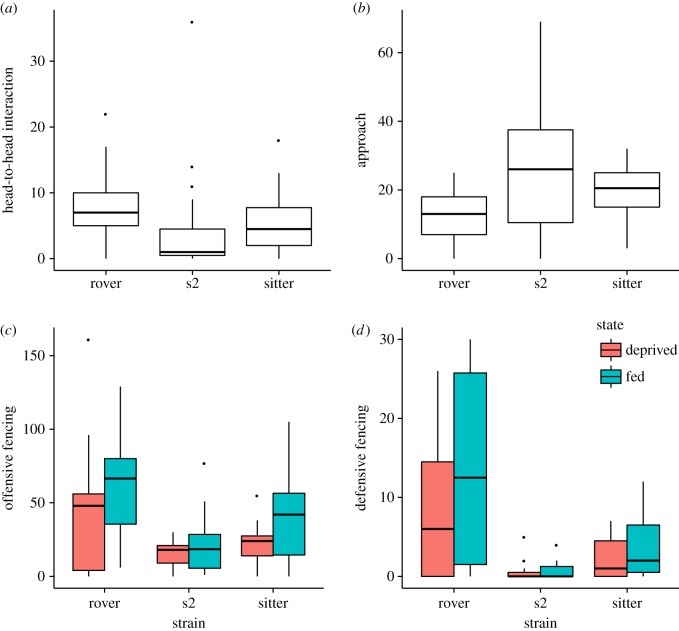
Boxplot of the frequency of head-to-head interaction (*a*), approach (*b*), offensive fencing (*c*) and defensive fencing (*d*) in fed and food-deprived rover, s2 and sitter male flies. Rover exhibited significantly more head-to-head interaction and fencing, but less approach than sitter and s2 males (*p*_(Bonferroni)_ < 0.05). Food deprivation did not have an effect on head-to-head interaction or approach, but it reduced offensive and defensive fencing (*p*_(Bonferroni)_ < 0.05; (*c,d*) the dots are outliers.

There was significant strain effect on all the aggressive behaviours except retreat (table 1). Rover pairs of flies exhibited more high- and intermediate-level aggressive behaviours: head-to-head interaction (figure 1*a*), lunging (electronic supplementary material, figure S1*c*), wing threat (electronic supplementary material, figure S1*b,d*), and fencing (figure 1*c,d*); but lower approach (figure 1*b*) than did sitter and s2 pairs.

**Table 1.** χ^2^ analysis of deviance of Poisson GLMs testing the effect of strain, food deprivation and their interaction on nine aggressive behaviours. The *p*-values that are below 0.05 after the Bonferroni correction are bolded. Rover, sitter and s2 differ in all the aggressive behaviours except retreat. Food deprivation influenced intermediate-level aggressive behaviours: fencing and wing threat, but did not influence other behaviours. There were significant interaction effects on lunging, chasing and offensive wing threat.

	strain effect			food deprivation effect			interaction effect		
behaviour	$\Delta \chi_{\left(d.f.=2\right)}^{2}$	*p*	*p*_Bonferroni_	$\Delta \chi_{\left(d.f.=1\right)}^{2}$	*p*	*p*_Bonferroni_	$\Delta \chi_{\left(d.f.=2\right)}^{2}$	*P*	*p*_Bonferroni_
head-to-head interaction	24.134	0.00001	**0.00005**	0.896	0.34390	1.00000	8.076	0.01763	0.15870
lunging	101.749	0.00000	**0.00000**	0.101	0.75017	1.00000	111.695	0.00000	**0.00000**
approach	100.480	0.00000	**0.00000**	2.551	0.11023	0.99203	7.558	0.02285	0.20562
chasing	11.718	0.00285	**0.02569**	0.003	0.95696	1.00000	36.193	0.00000	**0.00000**
offensive wing threat	530.351	0.00000	**0.00000**	128.577	0.00000	**0.00000**	49.580	0.00000	**0.00000**
defensive wing threat	201.492	0.00000	**0.00000**	207.264	0.00000	**0.00000**	1.331	0.51401	1.00000
offensive fencing	387.516	0.00000	**0.00000**	80.899	0.00000	**0.00000**	10.281	0.00585	0.05268
defensive fencing	247.996	0.00000	**0.00000**	19.740	0.00001	**0.00008**	0.224	0.89414	1.00000
retreat	2.143	0.34247	1.00000	4.797	0.02850	0.25652	1.447	0.48514	1.00000

For a collective view of offensive and defensive behaviours, the PC1 and PC2, respectively, explain 52.57 and 23.56% of the variation in offensive behaviours (figure 3*a*; electronic supplementary material, table S1) and 97.89 and 1.47% of the variation in defensive behaviours (figure 3*b*; electronic supplementary material, table S1). The level of aggressive behaviours was weighted negatively in PC1 of offensive behaviours, and weighted positively in PC2 of defensive behaviours (eigenvectors in figure 3*a,b*; electronic supplementary material, table S1). There was a significant effect of strain on PC1 (*F*_2, 60_ = 3.895, *p* = 0.026) and PC2 (*F*_2, 60_ = 4.583, *p* = 0.014) of offensive behaviours and PC2 of defensive behaviours: *F*_2, 60_ = 13.926, *p* < 10^−4^), where rover pairs performed more offensive (figure 3*c*) and defensive behaviours (figure 3*d*) than sitter and S2 pairs.

**Figure 2.**
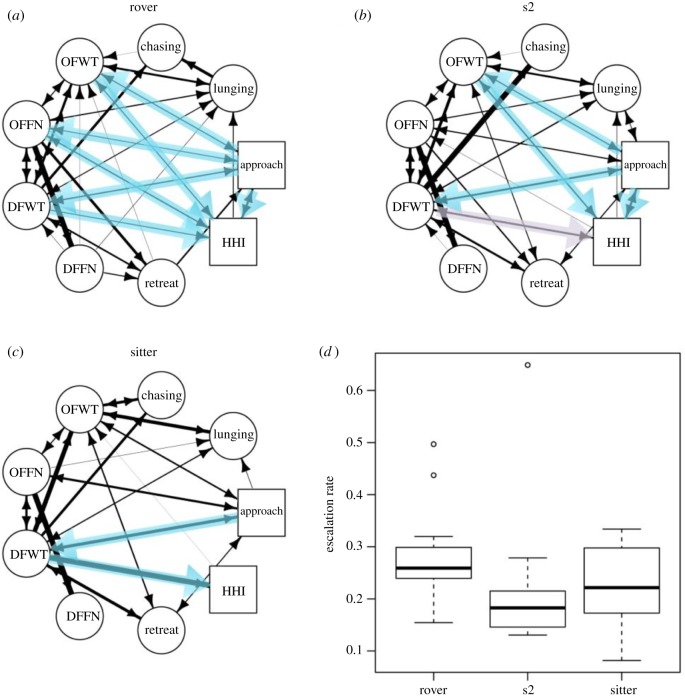
Averaged aggressive behaviour transition networks of rover (*a*), s2 (*b*) and sitter male flies (*c*), including directional transitions that occurred at least once. The width of each transition is proportional to the relative frequency of this transition over all transitions from the preceding behaviour. Short transition paths (two transitions or less) from approach to head-to-head interaction (HHI) that contain two steps or less were highlighted by blue shades. The purple-shaded arrow in s2 (*b*) from DFWT to HHI is rare compared with the highly likely alternative path from DFWT to chasing. (*d*) Boxplot shows escalation rate (transition rate from approach to HHI) variation across rover, s2 and sitter flies.

To further parse out the variation in fighting patterns among strains, aggressive escalation rates were estimated with behaviour transition networks (figure 2*a–c*). For each pair of strains, if the 95% confidence interval of the bootstrap escalation rate difference did not include zero, the escalation rates were significantly different. The 95% confidence intervals for the bootstrap escalation rate differences were 0.0034–0.1135 for rover versus sitter (rover > sitter), −0.0743 to 0.0704 for rover versus s2 and −0.0343 to 0.1496 for s2 versus sitter pairs (electronic supplementary material, figure S3). Thus, rovers significantly escalated more quickly than sitter flies, while s2 and sitter did not differ in the escalation pattern (figure 2*d*).

### 3.2. Food deprivation and body weight

We found a significant effect of strain (*F*_2, 84_ = 53.319, *p* < 10^−14^) and food deprivation treatment (*F*_1, 84_ = 100.622, *p* < 10^−15^), but not an interaction of the two factors (*p* > 0.05), on body weights of the tested flies. Rovers were lighter than sitter and s2; and food-deprived flies were lighter than fed flies (electronic supplementary material, figure S2*a*). Thus, the food deprivation treatment was sufficient to affect the weight of the flies.

### 3.3. Food deprivation and aggression

Although food deprivation was sufficient to affect body weight change, it did not elevate aggression. Food deprivation did not elevate aggressive behaviour; instead, it significantly reduced it (figure 1*c,d*; electronic supplementary material, figure S1*b,d*). Specifically, food deprivation did not influence PC1 or PC2 of offensive behaviour (*p* > 0.05), but it reduced the individual frequency of intermediate-level aggressive behaviours (wing threat and fencing, table 1 and figure 1*c,d*; electronic supplementary material, figure S1*b,d*). Fed flies exhibited more aggressive behaviours than food-deprived flies (figure 1*c,d*), which contradicted the prediction from hypothesis 2 that food-deprived flies would be more aggressive.

There was no strain–treatment interaction effect on most of the individual aggressive behaviours (*p* > 0.05) or on the offensive (*p* > 0.05) or defensive (*p* > 0.05) behaviours as a whole. The only behaviours that were significantly influenced by interaction were: lunging (table 1; electronic supplementary material, figure S1*c*), chasing (table 1; electronic supplementary material, figure S1*a*) and offensive wing threat (table 1; electronic supplementary material, figure S1*b*).

Food deprivation did not alter defensive behaviours or aggressive escalation rate. There was no effect of food deprivation, or an interaction between food deprivation and strain for the PC1 or PC2 of defensive behaviours (*p* > 0.05). In addition, no difference in escalation rate was observed for fed versus deprived flies, as the 95% bootstrap confidence interval of the mean difference between fed and food-deprived flies is −0.0253 to 0.0926 (electronic supplementary material, figure S3).

This reduction in offensive behaviour might reflect a general reduction in locomotion from the food deprivation treatment. However, this possibility is ruled out by that fact that there is no effect of food deprivation ($\Delta \chi_{\left(d.f.=1\right)}^{2}=0.017,$ *p* = 0.896, electronic supplementary material, figure S2*b*) on the activity score (the total frequency of both flies moving onto food). Although there is a significant strain–treatment interaction ($\Delta \chi_{\left(d.f.=2\right)}^{2}=27.246,$ *p* < 10^−5^) on the activity score, food deprivation did not decrease rover or sitter activity score, but increased rover activity score (electronic supplementary material, figure S2*b*).

## 4. Discussion

**Figure 3.**
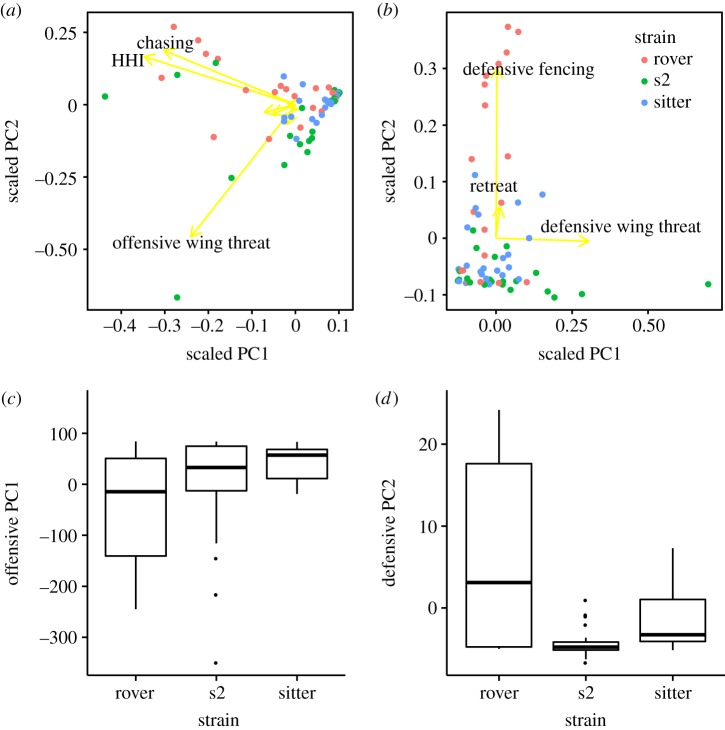
(*a,b*) PCA biplots of offensive (*a*) and defensive behaviours (*b*) in male rover, s2 and sitter flies. Rover, s2 and sitter flies are colour-code as shown in the legend. The yellow arrows represent the eigenvectors of behaviours, which show the relation between each behaviour and PC1 as well as PC2. (*a*) Head-to-head interaction (HHI), chasing and offensive wing threat demonstrate the most variation in offensive PCA, and are negatively weighted in PC1. Thus, lower PC1 reflects greater offensive behaviour intensity (*a*). (*b*) There are three defensive behaviours in defensive PCA, defensive fencing, defensive wing threat and retreat. (*c,d*) Boxplots show the effect of strain on the PC axes that represent variation in aggression (PC1 of offensive behaviours, *p* = 0.0257 (*c*); and PC2 of defensive behaviours, *p* < 10^−4^ (*d*)).

We found that *for* influences variation in the frequency of a variety of aggressive behaviours (figure 1), offensive and defensive behaviours as a whole (figure 3), and escalation patterns between aggressive encounters (figure 2). Furthermore, *for* appears to regulate aggression independent of its impact on the response to food deprivation. Behavioural genetic mechanisms of aggression and other *for*-regulated traits might comprise a co-adaptive complex arising from the pleiotropy of the *for* gene.

### 4.1. Neuromolecular underpinnings

Our finding of an effect of *for* on aggressive behaviour suggests potential interaction between *for* and molecular pathways that modulate aggression. cGMP-dependent protein kinase (PKG), the product of *for*, has higher enzyme activity in rover adult heads than in sitter [12]. The fact that rover is more aggressive than sitters indicates that PKG might elevate aggression. Consequently, PKG might interact with at least two neuromolecular pathways that are known to modulate aggressive behaviour: serotonin (5-HT) and octopamine. These two molecules are important neurotransmitters for fruit fly aggression [45,46]. PKG regulates 5-HT signalling through binding and activating serotonin transporter (SERT) in the presence of cGMP in mammals [47]. In addition, the octopamine system shares the targeting molecule, Na^+^-activated K^+^ (K/Na) channel, with cGMP/PKG in mushroom bodies of the cricket [48]. Thus, octopamine-mediated aggression signalling can be influenced by PKG activity. Taken together, the *for*-encoded PKG brain activity might directly interact with key neural modulators of aggressive behaviour, causing drastically different rover–sitter aggressive behaviour.

### 4.2. Food deprivation and aggression

Food deprivation was predicted to influence the aggression decision-making of the flies in resource competition, as it decreased the tendency to execute offensive behaviour. The short-term effect of food deprivation did not alter the escalation pattern, but it decreased intermediate-level aggressive behaviours (wing threat and fencing, table 1 and figure 1*c,d*; electronic supplementary material, figure S1*b,d*). This result is surprising, considering that optimal foraging theory predicts that animals take more risks when the perceived value of the resource is high [23]. Accordingly, flies should employ a risk-prone foraging strategy when they have been food-deprived for a significant amount of time, as the value of the food increases with starvation [49,50]. Nonetheless, elevated hunger level might increase the benefit or ‘value’ of the resources, but also increase the cost of aggression to defend food. In the light of Economic Defensibility Theory [51], resources are only defended if the benefit outweighs the cost, thus offensive execution will be suppressed if the perceived cost offsets the value of food acquisition.

In adult fruit flies, a 24 h food deprivation treatment is known to increase a fly's responsiveness to a drop of sucrose [20,52]. In the present study, the 24 h food deprivation treatment resulted in a significant reduction in body weight in each strain (electronic supplementary material, figure S2), but it did not increase individual aggressive behaviour levels as predicted. Instead, a reduction in the levels of intermediate aggressive behaviours such as wing threat and fencing was found (table 1 and figure 1*c,d*; electronic supplementary material, figure S1*d*). We might have expected a reduction in locomotion in general, which could have resulted in a decrease in offensive and defensive behaviours had 24 h of food deprivation been too long a period of deprivation. However, this was not supported because the food deprivation treatment did not change the total activity score of the flies (electronic supplementary material, figure S2*b*). In addition, we found a trend of decreasing aggressive escalation rate after food deprivation (electronic supplementary material, figure S3). Thus, the food deprivation treatment specifically suppressed aggressive motivation, potentially due to the perceived cost outweighing the benefit.

### 4.3. *for* pleiotropy and evolution

The pleiotropic effect of *for* might be universal, given that it influences vastly different traits and the functional association between phenotypes may offset the gene-to-phenotype effects. In particular, in addition to *for*'s effects on naturally selected traits such as food-related traits, learning and memory, and sleep (reviewed in [14]), we found that *for* affects aggression, a social behaviour that is usually sexually selected. The mutational effect of *for* can be antagonistic among these traits, as adaptation of certain traits might be compromised due to the adaptation of the others [3]. In fact, the inhibitory effect of food deprivation on offensive behaviour reflects antagonism among *for*-regulated traits. If so, such antagonism will limit the evolution of complexity [3].

Nevertheless, *for*'s pleiotropy could be modular if the molecular pathways underlying food-related traits and aggression converge, contributing to a large functionally connected trait repertoire. As discussed, the known neuromolecular underpinnings of aggression might interact with *for* through serotonin (5-HT) and octopamine pathways. Hence, the *for* effect could be confined in a gene–trait module, with mediators that alleviate regulatory conflicts, forming a synergistic complex that facilitates adaptation. Indeed, a genetic algorithm can evolve a modular network to simultaneously accommodate two very different functions [53]. The wiring of such a gene–traits module might ultimately give rise to evolvable ‘strategies’ (discussed above), selected and maintained by negative frequency-dependent selection.

### 4.4. Evolution of aggressive strategies

The difference between rover and sitter aggressive behaviour reflects alternative aggressive strategies that might be maintained by negative frequency-dependent selection. The crucial strategic decision of whether to escalate is made during aggressive encounters, which involve sequential transition from lower-level ‘locating’-oriented behaviour to higher-level (riskier) ‘contacting’-based behaviours [54]. Rovers employ an ‘offensive’ fighting strategy, displaying more offensive behaviours overall (figure 3) and faster transition from initial approach to physical holding, tussling and boxing (figure 2*a*); whereas sitter's aggressive strategy is ‘defensive’, demonstrating less aggressive behaviour (figure 3), and sitters are less likely to escalate the fight (figure 2*c*).

Aggressive and foraging behaviours can be co-selected in a cluster of co-adapted traits complex wired within the pleiotropic *for* gene. Rover and sitter alleles are maintained by negative frequency-dependent selection, forming bimodal adaptive peaks [17]. Aggressive and foraging behaviour might synergistically compose adaptive complexes underlying alternative strategies of rover and sitter for survival and reproduction. In particular, an aggressive strategy might be more compatible with a rover foraging strategy: if aggressive individuals can colonize novel food patches more easily, an offensive strategy can be selected for in rover foragers that tend to travel more when foraging. Rover-‘offensive’ and sitter-‘defensive’ aggressive strategies could potentially be maintained by negative frequency-dependent selection. When sitters are rare, the rover aggressive strategy is costly due to harmful fights with abundant rovers opponents. However, when rovers are rare, adult aggressive strategy can be beneficial, since it will dominate foraging and mating opportunities among ‘defensive’ sitters. Such aggressive rover parents could guard spacious larval food patches that favour rover larvae which tend to move around when foraging. This negative frequency-dependent co-adaptation of feeding and fighting strategies might occur across life-history stages and generations.

In fact, such alternative social strategies, characterized by behavioural and life-history trait combinations [55], are frequently seen in other taxa, such as white-throated sparrows [56] and side-blotched lizards [57]. Future study should test this idea by tracking fitness, aggression and foraging path length across life-history stages in populations of various rover-to-sitter frequencies.

## 5. Conclusion

The pleiotropic effect of *for* is broader than previously thought. In addition to various phenotypes that include learning, memory and sleeping, we explored *for* impact on aggressive strategies. The relationship between the *for* gene and aggression does not appear to be through *for*'s impact on the response to food deprivation, since food deprivation did not elevate offensive behaviour, but suppressed it. This unexpected pattern reflects agonistic effects between the pleiotropic gene-targeted traits. Aggressive behaviour might interact with other traits under the control of *for* and/or its molecular underpinning might converge with *for* molecular activities, forming a large gene–trait adaptive complex to alleviate functional conflicts. Future studies should examine pairwise functional association among *for* regulated traits, to address the functional architecture of the trait complex. This will reveal synergistic and/or discordant evolutionary forces that shape this powerful adaptive complex woven by a pleiotropic gene.

## Supplementary Material

Figure S1-S3 and Tables S1

## Data accessibility

All the raw data and code for analyses in the manuscript are publically available. The raw data for all the analysis have been deposited in Dryad: http://dx.doi.org/10.5061/dryad.41pr0 [44]. The custom code [41] for calculating aggression escalation rate has been deposited in github.
