# Supplementary material for: Aggressive behaviours, food deprivation and the foraging gene
Source: R Soc Open Sci. 2017 Apr 26;4(4):170042. doi: 10.1098/rsos.170042 (PMC5414267; doi:10.1098/rsos.170042)
Supplement: Figure S1-S3 and Tables S1 [file rsos170042supp1.docx]

**Supplementary Materials**

**Figure S1** Boxplots showing the effect of strain and a food-deprivation treatment on the frequency of aggressive behaviors: chasing (A), lunging (C), as well as offensive (B) and defensive wing threat (D). There was a significant strain effect on all the behaviors (*p* < 0.05). There was also a significant effect of food deprivation (*p* < 0.05) on wing threat (B, D). The interaction between strain and treatment significantly (*p* < 0.05) influenced the frequency of chasing (A), offensive wing threat (B), and lunging (C).

**Figure S2** Boxplots of dry body weight (A) and the activity score (B) among fed and food-deprived male rover, s2, and sitter flies. The dots above and below boxplots are outliers. (A), body weight significantly differed among strains, and was decreased by the food-deprivation treatment. (B), activity scores was measured as the total number of times that both males in the pair moved onto the food cup. Activity score was not influenced by the food-deprivation treatment (*p* > 0.05) but there was a significant interaction between strain and food deprivation treatment on the activity score (*p* < 10^-5^).

**Figure S3** Bootstrap distribution of mean escalation rate differences of rover-sitter (A), rover-s2 (B), s2-sitter (C) pairs as well as fed compared to food-deprived flies (D). Only the rover and sitter escalation rate difference significantly deviated from zero (95% interval: 0.0034 to 0.1135).

|  |  | PC1 | PC2 | PC3 | PC4 |
| --- | --- | --- | --- | --- | --- |
| Offensive behavior | Eigenvalues | 9778.6145 | 4381.7371 | 3141.8438 | 951.6564 |
|  | Proportion of variance | 0.5257 | 0.2356 | 0.1689 | 0.0512 |
|  | HHI.TN | -0.0343 | 0.0092 | -0.0060 | 0.0050 |
|  | HHI.TD | -0.6587 | 0.3153 | 0.6733 | 0.0938 |
|  | L.TN | -0.1351 | -0.0501 | -0.0072 | -0.0951 |
|  | AP.TN | -0.0357 | -0.1297 | -0.0024 | -0.0170 |
|  | Chasing.TN | -0.0313 | -0.0077 | -0.0214 | -0.0272 |
|  | Chasing.TD | -0.5725 | 0.3560 | -0.7117 | -0.1728 |
|  | Offensive.WT.TN | -0.4549 | -0.8664 | -0.0326 | -0.0973 |
|  | Offensive.Fencing.TN | -0.0985 | -0.0614 | -0.1964 | 0.9705 |
| Defensive behavior | Eigenvalues | 4182.1436 | 62.8807 | 27.2571 |  |
|  | Proportion of variance | 0.9789 | 0.0147 | 0.0064 |  |
|  | Defensive.WT.TN | 0.9991 | -0.0172 | 0.0380 |  |
|  | Defensive.fencing.TN | 0.0100 | 0.9832 | 0.1821 |  |
|  | retreat.TN | 0.0405 | 0.1815 | -0.9825 |  |

Table S1 Eigenvalues, proportion of variation, and loadings of the principal component analysis of offensive and defensive behaviors.
